# Sustained *Ex Vivo* Susceptibility of Plasmodium falciparum to Artemisinin Derivatives but Increasing Tolerance to Artemisinin Combination Therapy Partner Quinolines in The Gambia

**DOI:** 10.1128/AAC.00759-17

**Published:** 2017-11-22

**Authors:** Alfred Amambua-Ngwa, Joseph Okebe, Haddijatou Mbye, Sukai Ceesay, Fatima El-Fatouri, Fatou Joof, Haddy Nyang, Ramatoulie Janha, Muna Affara, Abdullahi Ahmad, Olimatou Kolly, Davis Nwakanma, Umberto D'Alessandro

**Affiliations:** a Medical Research Council Unit The Gambia, Fajara, The Gambia; b National Malaria Control Programme, Banjul, The Gambia; c London School of Hygiene and Tropical Medicine, London, United Kingdom

**Keywords:** alleles, antimalarial agents, artemisinin combination therapies, drug resistance evolution, *ex vivo* susceptibility, haplotypes, malaria elimination

## Abstract

Antimalarial interventions have yielded a significant decline in malaria prevalence in The Gambia, where artemether-lumefantrine (AL) has been used as a first-line antimalarial for a decade. Clinical Plasmodium falciparum isolates collected from 2012 to 2015 were analyzed *ex vivo* for antimalarial susceptibility and genotyped for drug resistance markers (*pfcrt* K76T, *pfmdr1* codons 86, 184, and 1246, and *pfk13*) and microsatellite variation. Additionally, allele frequencies of single nucleotide polymorphisms (SNPs) from other drug resistance-associated genes were compared from genomic sequence data sets from 2008 (*n* = 79) and 2014 (*n* = 168). No artemisinin resistance-associated *pfk13* mutation was found, and only 4% of the isolates tested in 2015 showed significant growth after exposure to dihydroartemisinin. Conversely, the 50% inhibitory concentrations (IC_50_s) of amodiaquine and lumefantrine increased within this period. *pfcrt* 76T and *pfmdr1* 184F mutants remained at a prevalence above 80%. *pfcrt* 76T was positively associated with higher IC_50_s to chloroquine. *pfmdr1* NYD increased in frequency between 2012 and 2015 due to lumefantrine selection. The TNYD (*pfcrt* 76T and *pfmdr1* NYD wild-type haplotype) also increased in frequency following AL implementation in 2008. These results suggest selection for *pfcrt* and *pfmdr1* genotypes that enable tolerance to lumefantrine. Increased tolerance to lumefantrine calls for sustained chemotherapeutic monitoring in The Gambia to minimize complete artemisinin combination therapy (ACT) failure in the future.

## INTRODUCTION

There are increasing reports on the persistence of Plasmodium falciparum parasitemia and elevated rates of reinfection following artemisinin combination therapies (ACTs) (1–5). These suggest that the therapeutic efficacy of ACTs might have been compromised beyond Southeast Asia, where artemisinin resistance has been confirmed (6, 7). Global vigilance against artemisinin resistance is therefore vital for sustaining successes toward malaria elimination, particularly in Africa, where malaria is still a significant health problem (7). Approaches to the routine surveillance of antimalarial therapeutic efficacy include *ex vivo* and molecular testing of P. falciparum isolates from natural populations (8). These assays determine the susceptibility of individual infections to several antimalarial drugs without the interference of human factors, such as dosing, compliance, immunity, and metabolism. Most P. falciparum *ex vivo* susceptibility assays report the concentration of antimalarial drugs that reduce parasite growth by half (50% inhibitory concentration [IC_50_]). Recently, the ring-stage survival assay (RSA) was developed to assess parasites' susceptibility to artemisinin (9, 10). With increased drug pressure from elimination interventions, they provide early indications of drug tolerance or developing resistance in parasite populations. Besides phenotypic assays, molecular surveys of drug resistance-associated genetic polymorphisms can be implemented in longitudinal samples of populations to monitor trends in mutant allele frequencies.

Mutations in the P. falciparum multidrug resistance (*pfmdr1*), chloroquine resistance transporter (*pfcrt*), dihydrofolate reductase (*pfdhfr*), and dihydropteroate synthase (*pfdhps*) genes have been widely described (11, 12). However, many mutations in other gene loci have been associated with drug resistance (13–16), including genes for multidrug resistance-associated proteins (*pfmrp1* and *pfmrp2*), calcium-transporting ATPase (*pfatp6*), multidrug resistance protein 2+ (heavy metal transport family) (*pfmdr2*), plasmepsin III, histo-aspartic protease (*pfhap*), sodium/hydrogen exchanger H^+^ antiporter (*pfnhe*), ubiquitin carboxyl-terminal hydrolase 1, putative (*pfubp1*), and Kelch propeller proteins (*pfk13*) (17). The *pfk13* gene is particularly important because single nucleotide polymorphisms (SNPs) on its propeller domain have been strongly associated with artemisinin resistance in Southeast Asia (18). It is not clear how the prevalences of these mutations have been affected by the substantial reduction in malaria transmission and sustained drug pressure in countries in sub-Saharan Africa, such as The Gambia (19–21).

The Gambia has achieved a substantial reduction in the malaria burden over the last 15 years, due to the high coverage of long-lasting insecticidal nets and prompt diagnosis and treatment with artemether-lumefantrine (AL) as a first-line ACT (19, 22). AL is procured centrally with support from the Global Fund and distributed to all public health facilities. However, AL, other ACTs, and antimalarials, such as chloroquine (CQ) and sulfadoxine-pyrimethamine (SP), are available from private pharmacies and the informal health sector. When AL was introduced in 2008, mutations related to SP resistance were almost at fixation, while the prevalence of mutations against quinolines, namely, *pfcrt* K76T and *pfmdr1* N86Y, were 60% and 30%, respectively (23). Though ACTs remain highly efficacious, high frequencies of nonsynonymous mutations on the K13 gene have been reported (24). In neighboring Senegal, other K13 mutations have emerged, and there has been an increase in the frequency of *pfmdr1* Y184F mutants associated with reduced sensitivity to artemisinin (25–27). With these reports and the lack of a readily deployable alternative to ACTs, continuous surveillance of polymorphisms and e*x vivo* testing in The Gambia and across the subregion are needed to detect trends that could further compromise ACT efficacy.

To determine the changes in the prevalences of drug resistance markers and their effect on antimalarial tolerance, we analyzed the *ex vivo* drug susceptibility phenotypes of P. falciparum isolates from western Gambia collected over four transmission seasons (2012 to 2015). The temporal patterns of drug resistance alleles and haplotypes were determined in these populations and from genomes of isolates collected in 2008 and 2014. Our results show that antimalarial drug resistance mutations remain at significant frequencies, while *ex vivo* susceptibilities to ACT partner drugs, lumefantrine (LUM) and amodiaquine (AMD), decreased over the period.

## RESULTS

### Drug susceptibility.

**FIG 1:**
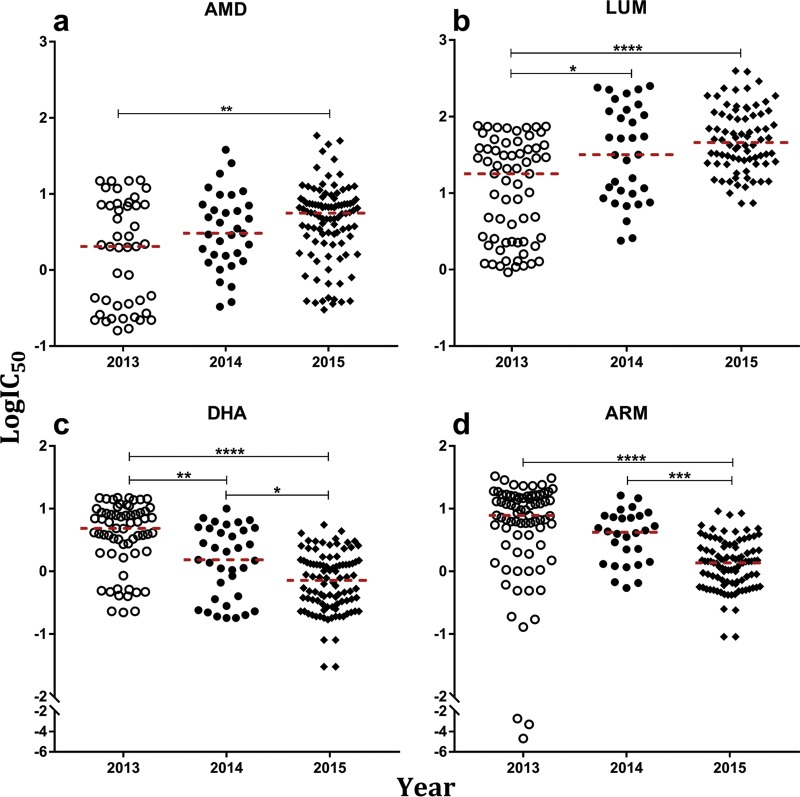
*In vitro* drug susceptibilities of Plasmodium falciparum isolates collected from Brikama (western Gambia) during the transmission seasons from years 2013 to 2015. Each plot shows the log_10_ of the 50% inhibitory concentration (logIC_50_) of isolates for a drug grouped per year of isolate collection as labeled on the *x* axes. Each point is the logIC_50_ for an isolate against amodiaquine (AMD) (a), lumefantrine (LUM) (b), dihydroartemisinin (DHA) (c), and artemether (ARM) (d). The median for each drug per year is shown as broken red lines. Lines connect pairs of years for which the distribution of IC_50_s were significantly different at a *P* value of <0.05 (*). The most significant differences had *P* values of <0.0001 (****).

We found SYBR Green-based fluorimetry or flow cytometry in combination with curve-fitting in IVART (28) to be reliable in estimating IC_50_s (see Table S1 and Fig. S1 in the supplemental material). With this approach, we successfully conducted 1,238 *ex vivo* antimalarial drug susceptibility (IC_50_) assays of P. falciparum by fluorimetry from 2012 to 2015 (Table 1). In malaria samples from 2013 to 2015, we consistently tested parasite isolates recruited during therapeutic efficacy studies against AMD, LUM, artemether (ARM), and dihydroartemisinin (DHA). We found significantly increasing IC_50_s for isolates from consecutive years against AMD (*P* = 0.0082) and LUM (*P* < 0.0001) (Fig. 1a and b and Table S2). Over the same period, there was a >3-fold decrease in the IC_50_s to the artemisinin derivatives ARM (*P* < 0.0001) and DHA (*P* < 0.0001) (Fig. 1c and d). The geometric mean of the IC_50_s between isolates from pairs of years was significantly different between successive years for all four drugs except for AMD, which was significant only between 2013 and 2015 (Table S3). There was a significant positive correlation in the IC_50_s to DHA and ARM (*P* < 0.0001) (Table 2). In contrast, there was a negative correlation between ARM and LUM IC_50_s (*P* = 0.00471). LUM IC_50_s were also negatively correlated with DHA, although not significantly (*P* = 0.2686). For isolates assayed in 2012, 2013, and 2014, the IC_50_s for mefloquine (MQ) did not change significantly (*P* = 0.3035) (Fig. S2a). However, the percentage of isolates with an IC_50_ beyond the 30 nM resistance threshold decreased from 71% to 54%.

**TABLE 1:** Number of clinical Plasmodium falciparum isolates recruited from Brikama, The Gambia, between 2012 and 2015 and assayed for *ex vivo* drug susceptibility or genotyped for microsatellite diversity and drug resistance-associated SNPs in *pfmdr1*, *pfcrt*, *pfatpase6*, and *pfk13*

Drug or genotyping	2012	2013	2014	2015	Total
Drugs					
Lumefantrine	33	65	33	78	209
Amodiaquine		45	33	100	178
Dihydroartemisinin		63	35	96	194
Artemether		76	28	96	200
Mefloquine	38	32	39		109
Artemisinin	46	34			80
Chloroquine	45			46	91
Quinine				59	59
Sulfadoxine				59	59
Pyrimethamine				59	59
Genotyping					
SNPs	78	125	67	101	371
Microsatellites		56	59	69	184

**TABLE 2:** Correlation between the IC_50_s against AMD, LUM, ARM, and DHA for clinical P. falciparum isolates analyzed between 2013 and 2015

Drug pair	*r*	*r*^2^	*P* value^*a*^	95% CI
AMD-LUM	0.078	0.0060	0.3668	−0.091 to 0.24
AMD-DHA	0.0076	5.817e-005	0.9277	−0.15 to 0.17
AMD-ARM	0.099	0.0098	0.2306	−0.063 to 0.26
ARM-LUM	−0.172	0.0295	**0.00471**	−0.33 to −0.0023
ARM-DHA	0.633	0.4007	**<0.0001**	0.528 to 0.718
LUM-DHA	−0.095	0.009	0.2686	−0.26 to 0.074

a Values in bold are significant *P* values for pairwise comparisons.

### Prevalence of isolates above resistance threshold.

**FIG 2:**
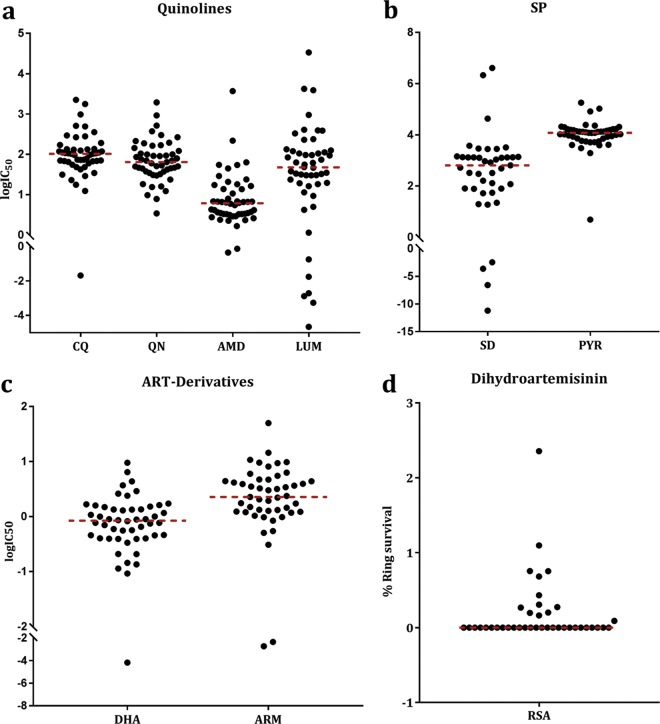
*In vitro* drug susceptibilities of Plasmodium falciparum isolates collected in the year 2015 from Brikama determined by flow cytometry (ACCURI) and IVART for standard inhibitory drug concentrations (IC_50_) and RSA against dihydroartemisinin. The logIC_50_ for each isolate is shown for quinolines (chloroquine [CQ], quinine [QN], AMD, and LUM) (a), SP drugs sulfadoxine (SD) and pyrimethamine (PYR) (b), and artemisinin derivatives DHA and ARM (c). (d) RSA. The percentage of infected cells with parasite growth after DHA treatment of ring stages for each isolate is shown. Broken red lines are the median value of the isolates for each assay.

To determine the prevalence of potentially resistant isolates in 2015, we analyzed 46 isolates for antimalarial susceptibility using a sensitive flow cytometry technique. We included CQ, quinine (QN), sulfadoxine (SD), and pyrimethamine (PYR) in our drug panel. Based on published *ex vivo* resistance thresholds, 43% of the isolates were resistant to CQ, 0% to QN, 15% to LUM, and 11% to AMD (Fig. 2a). Eighteen percent and 87% of the isolates had SD and PYR IC_50_s above 1 μM, respectively (Fig. 2b). Only one isolate had an IC_50_ above cutoff for the ART derivatives DHA and ARM (Fig. 2c). At a resistance threshold of 100 nM, the proportion of resistant isolates against CQ increased from 33% in 2012 to 43% in 2015, although there was no significant difference between the two populations (geometric means, 63.50 nM to 66.02 nM) (Fig. S2b).

### Ring survival of 2015 parasites.

**FIG 3:**
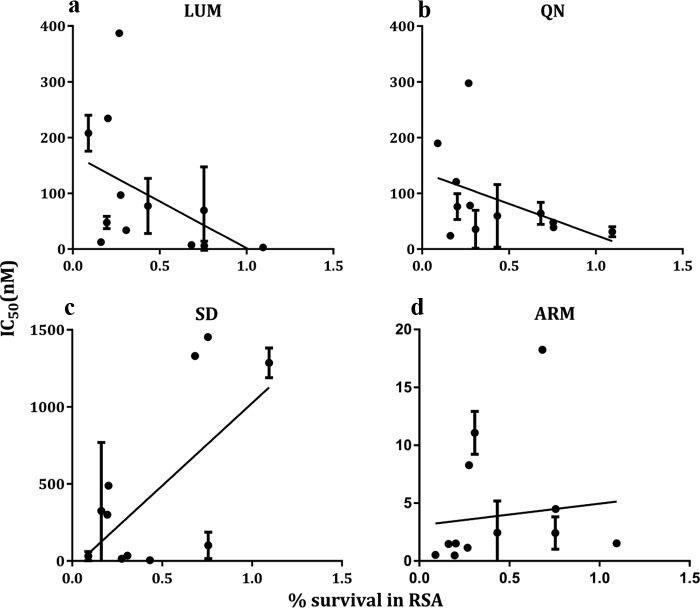
Correlation between drug IC_50_s (*y* axes) for LUM (a), QN (b), SD (c), and ARM (d) and the percentage of rings surviving after dihydroartemisinin exposure. Each point corresponds to the IC_50_ plotted against the percentage of infected cells with parasite growth for an isolate, with the confidence interval of the IC_50_ shown as bars.

Successful growth rates and percent survival of staggering-stage parasites following DHA exposure were achieved for 50 (73.5%) out of 68 isolates assayed by RSA in 2015. Parasite growth was found in 13 isolates (26%) after DHA exposure (Fig. 2d). The survival rate for isolates ranged from 0.05 to 2.5%. Only two (4%) isolates had a survival rate greater than 1%. There was a negative correlation between percent ring survival and the IC_50_s of LUM (*P* = 0.039, *r*^2^ = 0.196) and QN (*P* = 0.035, *r*^2^ = 0.186) (Fig. 3a and b). Conversely, a higher survival rate was seen for parasite isolates with higher IC_50_s against SD (*P* = 0.0038, *r*^2^ = 0.438) (Fig. 3c). There was also a positive correlation between survival rate and ARM IC_50_, but this was not significant (*P* = 0.565, *r*^2^ = 0.016) (Fig. 3d).

### Prevalence of drug resistance alleles.

**FIG 4:**
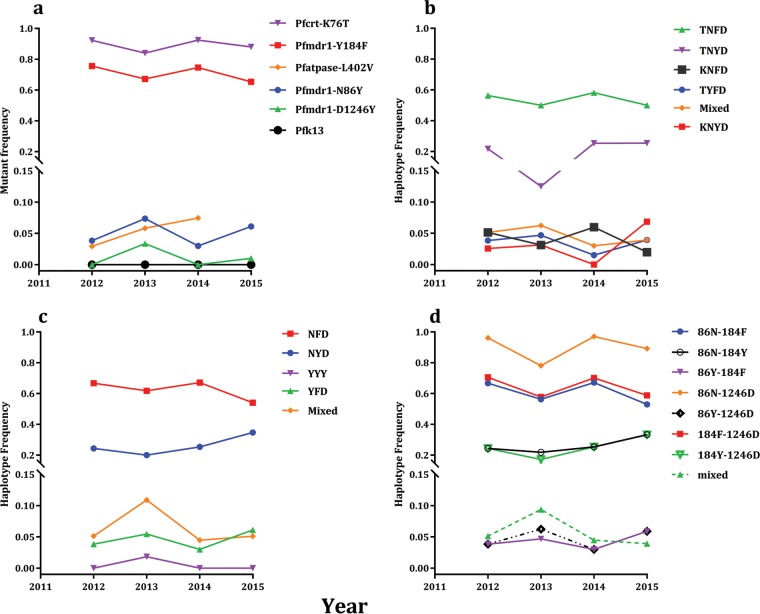
Temporal trends in the frequencies of Plasmodium falciparum drug resistance alleles at chloroquine resistance transporter (*pfcrt*), multidrug resistance protein 1 (*pfmdr1*), and calcium-transporting ATPase (*pfatpase6*) (a), *pfcrt-pfmdr1* haplotypes (b), *pfmdr1* codons 86-184-1246 haplotypes (c), and biallelic haplotypes at *pfmdr1* codons 86, 184, and 1246 (d). Isolates were collected across four transmission seasons (years 2012 to 2015) from Brikama, The Gambia. Each point represents the frequency (*y* axes) plotted against the year (*x* axes) isolates were collected. Each line shows the trend for frequencies of alleles or haplotypes in the figure legends.

Trends in *pfmdr1* and *pfcrt* drug resistance mutation frequencies were not consistent but fluctuated between years, with a rise in frequencies of *pfmdr1* 86Y and 1246Y mutants in 2013 and 2015 when there was a decline in *pfcrt* 76T and *pfmdr1* 184F compared to previous years (Fig. 4a). The mutant *pfmdr1* 86Y variant was present in <10% of the infections analyzed from 2012 to 2015 but showed a net increase within this period, although this was not statistically significant (*P* = 0.47152). Mutant *pfmdr1* 1246Y alleles were found in <1% of the isolates across years except for 2013, when they were present in 3% of the isolates. At least 70% of the isolates carried the *pfmdr1* 184F and *pfcrt* 76T mutant variants. There was a small insignificant decrease in the prevalence of infections with these variants (*pfmdr1* 184F and *pfcrt* 76T) in 2015 compared to 2012 (*P* = 0.13622 and 0.34212, respectively). The prevalence of the 76T mutant allele was lowest (82%) in 2013. No isolates were found with mutant alleles at the four *pfK13* codons (Y493H, R539T, I543T, and C580Y) associated with the artemisinin delayed clearance phenotype. Prior to the publication of the *pfK13*-associated mutations, we had genotyped L402V of *pfatpase6*, and this showed a 3-fold increase in prevalence from 2012 to 2014 (2.94 to 7.45%).

The most common haplotype for the four codons in *pfcrt* and *pfmdr1* combined was TNFD, incorporating mutant alleles at *pfcrt* codon 76 and *pfmdr1* codon 184. Its frequency fluctuated between years, but it remained at above 50% in 2015. For the years that there was an increase in the prevalence of TYFD, KNYD, and mixed haplotypes, there was a decline in the frequency of the highly prevalent TNFD, TNYD, and KNFD variant haplotypes (Fig. 4b). No triple *pfmdr1* mutants were found at codon 86, 184, or 1246. The frequency of the NFD haplotype of *pfmdr1*, which has a mutant variant only at codon 184, fluctuated between alternate years, with a net decline of 13% between 2012 and 2015 (Fig. 4c). The wild-type haplotype (NYD) gained a net increase in prevalence of 11% between 2012 and 2015, but this was not significant (Fig. S3). The most prevalent two-locus haplotype of *pfmdr1* was ND at codons 86 and 1246, present in at least 80% of the isolates genotyped across years (Fig. 4d). This was followed by NF (codons 86 and 184) and FD (codons 184 and 1246). The prevalence of these 3 haplotypes increased or declined in alternate years, but there was a net decline of 11% in prevalence between 2012 and 2015. The wild-type NY haplotype (codons 86 and 184) increased by 10% within the same period. The double mutant (YF) for *pfmdr1* codons 86 and 184 was present at 4% in 2012, and this increased to 6% in 2015. Other double-mutant combinations of the three codons were present only transiently and below 2%. Chi square and permutation tests of the differences in proportions of these haplotype variants between years were insignificant.

**FIG 5:**
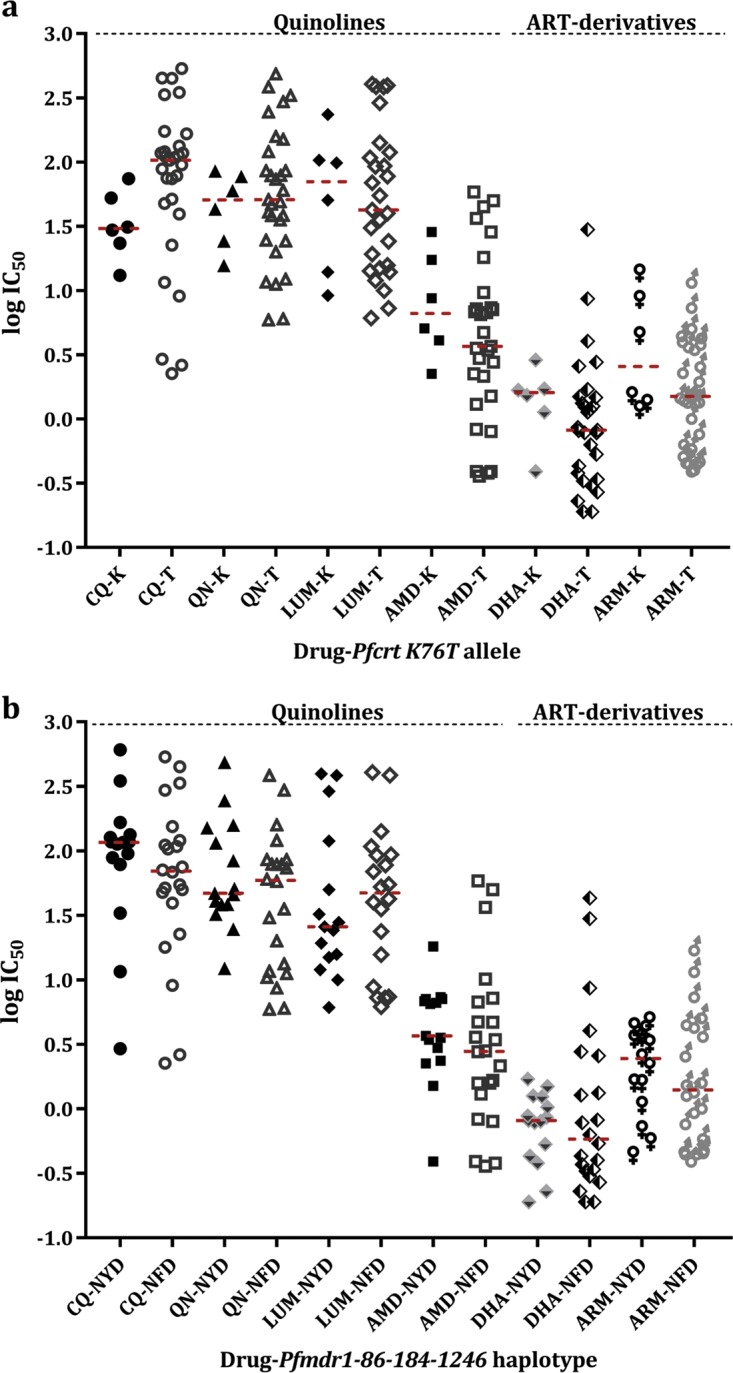
*In vitro* sensitivity (IC_50_) of Plasmodium falciparum isolates collected in the year 2015 for mutant or wild-type alleles at *pfcrt* K76T codon (a) and *pfmdr1*-86-184-1246 haplotypes of isolates assayed against quinolines (CQ, QN, LUM, and AMD) and artemisinin derivatives (DHA and ARM) (b). Each point in a group represents the logIC_50_ for an isolate with the specific allele or haplotype and drug labeled on the *x* axes. Broken red lines show the median logIC_50_ values for each haplotype group per drug.

There was a significant increase in IC_50_s to CQ for isolates that had the 76T mutation at the *pfcrt* codon 76 (*P* = 0.000, Fig. 5a). Lower IC_50_s were observed for 76T mutants against QN, AMQ, and LUM, although these were nonsignificant. There was a wide range of IC_50_s for isolates with either the mutant (NFD) or wild (NYD) *pfmdr1* haplotype against all drugs (Fig. 5b). Compared to NYD, the IC_50_s of isolates with the NFD haplotype were nonsignificantly higher against QN and LUM but lower against CQ and AMD. There was no significant association between the *pfcrt* or *pfmdr1* haplotypes and the IC_50_s for ART derivatives.

### Frequencies of drug resistance loci from genomes of 2008 and 2014 isolates.

The allele frequencies of SNPs in drug resistance-associated genes determined from publicly available genomes sequences of P. falciparum isolates collected from The Gambia in 2008 and 2014 were generally below 5% in both populations. However, 18 polymorphisms showed an increase in nonreference allele frequencies in 2014 isolates compared to 2008 isolates (Table S4). These included 2 polymorphisms in ABC transporters (*pfmrp2*), 5 polymorphisms in calcium-transporting ATPase (*pfatp6*), 1 polymorphism in multidrug resistance protein (*pfmdr1*), 2 polymorphisms in multidrug resistance protein 2+ (heavy metal transport family) (*pfmdr2*), 2 polymorphisms in multidrug resistance-associated protein 1+ (*pfmrp1*), 1 polymorphism in plasmepsin III, histo-aspartic protease (*pfhap*), 5 polymorphisms in sodium/hydrogen exchanger, Na^+^, H^++^ antiporter (*pfnhe*), and 3 polymorphisms in ubiquitin carboxyl-terminal hydrolase 1, putative (*pfubp1*). These allele frequency shifts were small, and only five of the analyzed SNPs attained a frequency of 5% or higher in the 2014 population: *pfatpase6* N569K (0.073 to 0.08), *pfmrp2* A1643V (0.01 to 0.05), and *pfnhe* H1401Q (0.03 to 0.05). *pfnhe* I1480K and S207F mutations were not detected in the 2008 population but were present at 5% and 10%, respectively, of the isolates from 2014.

### Parasite population structure.

The level of microsatellite heterozygosity was similar across all populations (2013 to 2013), with a mean of 0.813 (standard deviation, 0.049; 95% confidence interval [CI], 0.714 to 0.885). The genetic distances between the three yearly populations determined by Weir and Cockerham's index of differentiation (Fst) were insignificant between all population pairs (Table S6). The three populations showed no clustering by year of sampling based on the alleles of the six microsatellite loci genotyped. However, there were three subclusters determined by both axes 1 and 2 of principal components, with memberships constituting isolates from all three time points (Fig. S4).

## DISCUSSION

Using clinical Plasmodium falciparum isolates collected through three transmission seasons (2013 to 2015), we found increasing *ex vivo* tolerance to ACT partner drugs (LUM and AMD) and a high prevalence of known drug resistance alleles in *pfmdr1* and *pfcrt* 6 years after the introduction of AL in The Gambia (West Africa). Such a longitudinal analysis serves to provide an early warning on any compromise in the efficacy of ACTs. Our results are particularly relevant for countries, such as The Gambia, where ACTs have been in use for a decade, and the last *ex vivo* drug susceptibility testing of P. falciparum in this population was done 17 years ago, prior to their official introduction (29). Moreover, The Gambia is bordered on three sides by Senegal, which uses a different ACT artesunate-amodiaquine (ASAQ), and has reported increasing *ex vivo* tolerance to AMD (30). Amodiaquine is not officially recommended for the treatment of malaria in The Gambia, but human population movement between these countries could facilitate an exchange of drug-resistant parasites. Our results are also in line with reports from other West African countries of sustained *ex vivo* resistance of local P. falciparum populations to multiple quinolines that are not officially in use (31). While this could partly be due to cross-resistance between quinolines, such as CQ and AMD, access to these drugs from the informal/private sector could be selecting for less-susceptible parasites (32). Indeed, the IC_50_s for CQ determined in 2013 and 2015 in The Gambia remained high above the resistance cutoff despite a 2-fold reduction in median values within this period. For 39 isolates assayed in 2014, MQ resistance was still at 54% but down from 71% in the previous year. These results suggest closer monitoring of quinolines, as sustained drug pressure and multidrug-resistant P. falciparum could limit options for ACTs that can be officially deployed (33).

Lumefantrine in combination with ARM is the most widely used ACT in The Gambia and most of sub-Saharan Africa. While AL remains highly efficacious in The Gambia, an increasing proportion of patients with parasitemia between the 3rd and 28th days after treatment have been observed (Gambian antimalarial therapeutic efficacy studies in 2012 to 2014, our unpublished data). This is consistent with our finding of increasing IC_50_s to LUM in the same period. Lumefantrine resistance is a major threat to the efficacy of AL-based ACT, since evidence of rapid selection against it was reported in isolates with no prior exposure to ACTs (34). The parasite populations analyzed here showed no genetic differentiation but sustained susceptibility to the artemisinin derivatives ARM and DHA. This sustained susceptibility was confirmed by the results of an RSA conducted for the 2015 samples, which showed only 2 out of 50 isolates as having a greater than 1% survival rate after DHA exposure. Interestingly, we observed an increased survival rate against DHA for isolates with higher SD IC_50_s. Folate pathways are important for cell survival, and hence, we hypothesize that isolates that could salvage folates in the presence of SD could survive better against DHA. This finding will require further investigation, especially as selective pressure from the use of SP in season malaria chemoprophylaxis (SMC) could encourage DHA tolerance. However, we found none of the *pfK13* polymorphisms associated with artemisinin resistance in the four populations genotyped. These results together support the continuous use of AL in The Gambia, without excluding future considerations for a change in ACT regimen if LUM tolerance increases.

The choice of ACT alternatives could be guided by the prevalence of polymorphisms associated with antimalarial drug resistance in the population. We showed here that the major CQ resistance mutation *pfcrt* 76T remained highly prevalent in current isolates from The Gambia. Its slow temporal decline contrasts with reports in Kenya and Malawi, where the dramatic return of the sensitive genotype sparked reconsideration of CQ for malaria treatment (35, 36). We also found high frequencies of 86N and 10% of isolates with 1246D, which lead to higher *in vitro* and *in vivo* tolerance and reinfection rates after AL treatment (37). These were linked in the dominant 86N/184F/1246D *pfmdr1* haplotype found in isolates with higher IC_50_s to LUM. This haplotype had been shown to resist 15-fold the serum concentration of clinical doses of LUM (38). AL also selects for other *pfmdr1* haplotypes, like the NYD, which showed a steady increase in frequencies between temporal populations (39). This is consistent with studies in West Africa that found selection of these haplotypes by AL (40–42). Considering the risk to AL efficacy, an extended study of the temporal trends and association of these alleles with drug efficacy as parasite prevalence reduces could guide policy on ACTs in The Gambia. Taking advantage of publicly available genomic data sets from 2008 and 2014, we found increased frequencies of SNPs in a number of drug resistance-associated genes, suggesting a need for sustained vigilance as drug interventions continue. Sustained pressure from quinolines are maintaining *pfcrt* resistance variants. Hence, the widely proposed ACT regiments, such as DHA-piperaquine, will not be a good option, as piperaquine selects for CQ-resistant parasites.

The parasite population studied here has been in a decline for over a decade, and we prognosed that inbreeding could favor the rapid establishment of introduced or emerging drug-tolerant strains. The temporal populations showed no substructuring, as expected across Africa. The levels of heterozygosity at neutral microsatellite loci were comparable across the temporal populations and to those in previous reports (43). This high heterogeneity and lack of structure should disfavor easy establishment of new drug resistance mutants, as recombination remains significant.

This study combines molecular and phenotypic analyses to provide an insight into drug susceptibility trends and possible mechanisms determining patterns of susceptibility as ACTs continue to be widely implemented in The Gambia. It has shown artemisinin derivatives to be continuously potent against P. falciparum isolates from West of The Gambia. There is, however, an increase in *ex vivo* tolerance to the ACT partner drug lumefantrine. Levels of the *pfmdr1* 184F and *pfcrt* 76T mutants remain high, while there are emerging mutations in *pfmrp2* and *pfnhe* that suggest continuous selection from quinoline-based antimalarials. These results call for continuous and rigorous surveillance to sustain the useful life span of currently employed ACTs in The Gambia and other regions endemic for malaria.

## MATERIALS AND METHODS

### Consent and sample collection.

The study design and protocols were approved by the Gambian Government/MRC Joint Ethics Committee. Study subjects (age 6 months to 15 years) were recruited from the Brikama Health Centre, located in western Gambia, as part of therapeutic efficacy studies conducted during the 2012-2015 rainy (transmission) seasons (September to December). Inclusion criteria were a history of fever in the preceding 24 h, P. falciparum monoinfection at a parasite density of >1,000/μl of blood, as determined by microscopy, and no symptoms of complicated/severe malaria. Caregivers provided a written informed consent after the procedures were explained, and children between 12 and 15 years provided an assent in addition. A 2.5-ml blood sample was collected into EDTA tubes (BD Biosciences, Germany) and transported on ice (4°C) within 4 h of collection to the MRC, The Gambia (MRCG) laboratories for analysis. Following centrifugation, plasma and the buffy coat were recovered and stored frozen. RPMI 1640 medium (Sigma-Aldrich, UK) was added to the remaining cell pellet to obtain a 50% cell suspension, which was then overlaid on 3 ml of NycoPrep for leukocyte depletion (Axis Shield, UK). Leukocytes (peripheral blood mononuclear cells [PBMCs]) were separated following centrifugation at 2,500 rpm for 10 min with no break. The red blood cell (RBC) pellet was washed three times with RPMI 1640 medium and resuspended in growth medium at 2% hematocrit for drug susceptibility assays.

### *Ex vivo* drug susceptibility assays.

Laboratory-maintained P. falciparum strains (3D7, DD2, HB3, K1, and W2) were first assayed against lumefantrine (LUM), dihydroartemisinin (DHA), artemisinin (ART), and artesunate (ARS) to compare the consistencies of fluorimetric and cytometric determinations of parasite growth. We compared fitted curves between methods using GraphPad Prism and IVART. Isolates from 2013 to 2015 were then tested in triplicate on a panel of drugs composing ACTs, i.e., LUM, DHA, artemether (ARM), and amodiaquine (AMD), donated by the Worldwide Anti-Malarial Resistance Network (WWARN). The following drugs with historically reported antimalarial resistance were also tested: CQ in 2012, 2013, and 2015; quinine (QN) and mefloquine (MQ) in 2013 and 2014; and pyrimethamine (PYR) and sulfadoxine (SD) in 2015. The final concentration ranges tested were 750 to 3 nM for CQ, 1,500 to 6 nM for QN, 300 to 1.2 nM for MQ, 2,000 to 8 nM for LUM, 100 to 0.4 nM for AMD, 150 to 0.6 nM for ARM and DHA, 2,600 to 10 nM for SD, and 295,000 to 3 nM for PYR. Samples or control isolates (P. falciparum 3D7, DD2, and W2) were tested at a parasitemia of 0.5 to 1% in 2% hematocrit. Smears of the drug-free wells were observed by microscopy at the end of this period to ensure that the parasite strains had grown through a full cycle. For SYBR Green staining of parasite DNA, the cell pellet of each assay was suspended in 100 μl of SYBR Green I (1:10,000 dilutions in phosphate-buffered saline [PBS]) (Invitrogen, USA) and incubated in the dark for 1 h. Stained cells were washed in RPMI and resuspended in 100 μl of filtered PBS, and 100,000 events were acquired on an Accuri flow cytometer (BD). Flow cytometry cell counts were analyzed with the FlowJo software (Tree Star, Inc.). For fluorimetric determination of parasitemia on a Fluoroskan, 50 μl of stained cells was lysed in an equal volume of SYBR Green lysis buffer containing 0.005% SDS and read at 450 nm.

Cell counts were fitted with GraphPad Prism version 6 by a nonlinear regression against the logarithm of the drug concentration, using the formula for sigmoidal dose-response (variable slope) to yield the dose-response curve and resultant 50% inhibitory concentration (IC_50_). The mean, median, and geometric mean of the IC_50_s were calculated for all populations. For validation of the IC_50_ results, data were also submitted online for fitting using the WWARN IVART software for all samples from each year. Agreement between the GraphPad and IVART results was assessed using Bland-Altman analysis. Nonparametric statistical tests in Prism and R were used to compare IC_50_ data between the transmission seasons.

### RSA.

For 2015, 68 isolates were tested against DHA by a ring-stage survival assay (RSA). For the RSA control, the P. falciparum isolates 1241 (DHA resistant) and 1239 (DHA sensitive) were analyzed together with the population samples. Assays for each lymphocyte-depleted infected RBC sample were set up in duplicates at 0.5 to 1% parasitemia and 2% hematocrit, as described in published protocols (10). Parasites were exposed for 6 h to 700 nM DHA in complete culture RPMI medium (cRPMI) and 0.1% dimethyl sulfoxide (DMSO) as a test control. Exposure was under established culture conditions (37°C in the presence of 5% O_2_, 5% CO_2_, and 90% N_2_). Following this, the RBC pellets were recovered, washed once in incomplete RPMI, and cultured for another 66 h in drug-free culture medium. Thin blood smear slides were prepared for each duplicate test and stained with Giemsa. Parasite survival rates were determined using the parasitemia at 0 h (before exposure), the parasitemia of the DMSO control (NE), and DHA-exposed tests at 72 h. The growth rate for each isolate was determined as the ratio of the nonexposed (NE) against the initial parasitemia. For each isolate with a growth rate of 1% or higher, the parasite percent survival was calculated as (growth in DHA/growth in NE) × 100.

### Microsatellite genotyping.

To determine the population structure, selected SNPs and six genomic microsatellite loci (Polyα, PK2, TAA81, ARA2, G377, and TAA87) were genotyped as described in previous studies (43). Fragment sizes were checked using GeneMapper4.1 (Life Technologies software). Further binning and size scoring were done with GeneMarker version 1.85 (SoftGenetics) to include peaks with a relative fluorescent intensity (RFU) of >100. Multilocus alleles for each sample were normalized and corrected using the Tandem 2 software. Genetic distances and diversity for the populations were determined in the Microsatellite Analyzer package.

### Drug resistance marker genotyping.

Samples were analyzed for molecular markers of drug resistance, including *pfcrt* K76T, *pfmdr1* N86Y, Y184F, and D1246Y, *pfatpase6* L402V and E431K, and four K13 propeller domain polymorphisms (493, 539, 540, and 580). Nucleotide alleles at SNPs determining these mutations were assayed by allelic discrimination using 20× TaqMan assay mixes with probes targeting wild and mutant SNPs labeled with 6-carboxyfluorescein (FAM) and VIC, respectively. Primers and probes for the four K13 SNPs were custom designed and sourced from Metabion (Table S5). DNA from each isolate was assayed in a 15-μl 1× universal TaqMan PCR master mix on the Bio-Rad CFX96 real-time thermocycler. Clustering of samples and allele scoring were achieved using the Bio-Rad CFX96 manager software. A subset of samples (10%) with wild-type and mutant alleles were subjected to confirmatory analysis by Big Dye version 3.1 sequencing on the 3130xl genetic analyzer. Allele frequencies for the targets were analyzed using the Stata software. The *pfcrt* K76T mutation was assayed by high-resolution melt analysis using allele-specific probes in the Roche LightCycler. Genotypes of P. falciparum from genome sequences of isolates collected from Brikama in 2008 and 2014 were genotyped for haplotypes of *pfcrt* and *pfmdr1*. The derived alleles of SNPs in the following drug resistance-associated loci were also determined: ubiquitin carboxyl-terminal hydrolase 1 (PF3D7_0104300), putative multidrug resistance-associated protein 1 (PF3D7_0112200), bifunctional dihydrofolate reductase-thymidylate synthase (PF3D7_0417200), drug/metabolite transporter, putative (PF3D7_0715800), drug/metabolite transporter, putative (PF3D7_0716900), hydroxymethyldihydropterin pyrophosphokinase-dihydropteroate synthase (PF3D7_0810800), multidrug resistance-associated protein 2 (PF3D7_1229100), sodium/hydrogen exchanger, Na^+^, H^+^ antiporter (PF3D7_1303500), ferredoxin, putative (PF3D7_1318100), kelch protein K13 (PF3D7_1343700), multidrug resistance protein 2 (PF3D7_1447900), and plasmepsin III (PF3D7_1408100).

### Statistical analyses.

GraphPad Prism and R softwares were used for all statistical analyses. For population comparison of the drug IC_50_s, differences between population means were compared by the Kruskal-Wallis test. The threshold for significant differences between years was set at 0.05 and determined by one-way analysis of variance (ANOVA). Dunn's multiple comparison was employed to correct for differences between population pairs. Proportions of isolates between populations with IC_50_s higher than a resistance threshold with mutant alleles or haplotypes were compared using a permutation test in R. Simple linear regression fitting was used to determine the significance of trends in allele or haplotype frequencies.

## Supplementary Material

Supplemental material
