# Supplementary material for: Sustained Ex Vivo Susceptibility of Plasmodium falciparum to Artemisinin Derivatives but Increasing Tolerance to Artemisinin Combination Therapy Partner Quinolines in The Gambia
Source: Antimicrob Agents Chemother. 2017 Nov 22;61(12):e00759-17. doi: 10.1128/AAC.00759-17 (PMC5700332; doi:10.1128/AAC.00759-17)
Supplement: Supplemental material [file supp_61_12_e00759-17__index.html]

Supplemental material 

# Sustained *Ex Vivo* Susceptibility of Plasmodium falciparum to Artemisinin Derivatives but Increasing Tolerance to Artemisinin Combination Therapy Partner Quinolines in The Gambia

## Supplemental material

- Supplemental file 1 -

  Supplemental Tables S1 to S6 and Figures S1 to S4

  PDF, 982K
