# Supplementary material for: Sustained Ex Vivo Susceptibility of Plasmodium falciparum to Artemisinin Derivatives but Increasing Tolerance to Artemisinin Combination Therapy Partner Quinolines in The Gambia
Source: Antimicrob Agents Chemother. 2017 Nov 22;61(12):e00759-17. doi: 10.1128/AAC.00759-17 (PMC5700332; doi:10.1128/AAC.00759-17)
Supplement: Supplemental material [file AAC.00759-17_zac012176712s1.pdf]

Supplementary table 1: Correlation of IC50 values determined for *Plasmodium falciparum* laboratory strains (3D7, HB3, DD2, K1, and w2) against lumefantrine (LUM), artemisinin (ART), artesunate (ARS) and dihydroartemisinin (DHA) using flow cytometry (FACS-ACCURI) or fluorimetry with SyBr Green staining (Fluoroskan_SyrGreen) or DAPI staining (Fluoroskan-DAPI). Each value is the correlation coefficient (r^2) for IC50 determined replicate assays per isolate for each drug. IC50 were determined from sigmoidal fitting of infected cell counts acquired by each method for each drug concentration. These results showed FACS and Fluorimetry with SyBr-Green stained erythrocytes to be more reliable for in vitro analysis of drug susceptibility for IC_{50}.

Isolate	Drug	FACS-ACCURI	Fluoskan-SyBrGreen	Fluoskan-DAPI
**3D7**	**LUM**	0.98	0.73	0.66
	**ART**	0.94	0.90	0.60
	**ARS**	0.98	0.61	0.90
	**DHA**	0.99	0.70	0.25
**HB3**	**LUM**	0.96	0.97	0.66
	**ART**	0.94	0.87	0.77
	**ARS**	0.98	0.89	0.72
	**DHA**	0.94	0.86	0.90
**DD2**	**LUM**	0.99	0.91	0.63
	**ART**	0.97	0.95	0.50
	**ARS**	0.99	0.94	0.72
	**DHA**	0.99	0.95	0.83
**K1**	**LUM**	0.96	0.89	0.93
	**ART**	0.95	0.94	0.44
	**ARS**	0.95	0.94	0.85
	**DHA**	0.97	0.96	0.71
**w2**	**LUM**	0.99	0.95	0.78
	**ART**	0.92	0.78	0.92
	**ARS**	0.98	0.95	0.94
	**DHA**	0.95	0.96	0.77

Supplementary table 2: Geometric mean and ranges of the 50% inhibitory drug concentrations (nM) of ACT partner drugs; Amodiaquine (AMD), Artemether (ARM), DHA and LUM, determined by *ex vivo* assays of parasite isolates collected in Brikama during the malaria transmission seasons of 2013, 2014 and 2015. The ranges are shown in parentheses below each geometric mean.

**Drugs**	**2013**	**2014**	**2015**
**AMD**	1.66<br>(1.03-2.68)	3.54<br>(2.29-5.45)	3.84<br>(3.08-4.78)
**LUM**	10.64<br>(7.32-15.46)	58.13<br>(36.79-91.85)	32.97<br>(26.31-41.31)
**DHA**	3.67<br>(2.69-4.99)	1.61<br>(1.0-2.59)	0.69<br>(0.54-0.87)
**ARM**	4.76<br>(2.69-8.26)	3.761<br>(2.53-5.58)	1.11<br>(0.96-1.29)

Supplementary table 3: Significance levels for differences in the median IC_{50} concentrations for isolates tested between 2013 and 2015 from Brikama. Column labels show the years compared. The median IC_{50} values for LUM, DHA and ARM were significantly different between consecutive years. For AMD the median was significantly different only between 2013 and 2015 with higher values in 2015.

**Drugs**	**2013/2014**	**2013/2015**	**2014/2015**
**AMD**	0.0836	0.0078	0.3874
**LUM**	0.0013	<0.0001	0.0188
**DHA**	0.0004	<0.0001	0.0041
**ARM**	0.0070	<0.0001	<0.0001

Supplementary table 4: Allele frequencies of drug resistance polymorphism that show an increase in isolates collected in 2014 compared to 2008. Positions of polymorphism are shown as SnpName, MutName presents the amino acid and codon change while the derived allele frequencies (DAF) for the two populations (Brikama 2008 and 2014) are shown as DAF_BK08 and DAF_BK14 respectively.

SnpName	Gene Description	MutName	DAF_BK08	DAF_BK14
Pf3D7_01_v3:192084	ubiquitin carboxyl-terminal hydrolase 1, putative (UBP1)	D606N	0.024193548	0.032258065
Pf3D7_01_v3:192424	ubiquitin carboxyl-terminal hydrolase 1, putative (UBP1)	N719S	0.008064516	0.024193548
Pf3D7_01_v3:196945	ubiquitin carboxyl-terminal hydrolase 1, putative (UBP1)	R2226K	0	0.016129032
Pf3D7_01_v3:266935	calcium-transporting ATPase (ATP6)	H747Y	0	0.016129032
Pf3D7_01_v3:267467	calcium-transporting ATPase (ATP6)	N569K	**0.072580645**	**0.080645161**
Pf3D7_01_v3:267847	calcium-transporting ATPase (ATP6)	D443N	0.008064516	0.024193548
Pf3D7_01_v3:268447	calcium-transporting ATPase (ATP6)	H243Y	0.008064516	0.032258065
Pf3D7_01_v3:269055	calcium-transporting ATPase (ATP6)	Y40F	0	0.016129032
Pf3D7_01_v3:467092	multidrug resistance-associated protein 1+(MRP1)	K789N	0.008064516	0.032258065
Pf3D7_01_v3:468495	multidrug resistance-associated protein 1+(MRP1)	A1257V	0.008064516	0.032258065
Pf3D7_05_v3:959401	multidrug resistance protein (MDR1)	N504K	0	0.032258065
Pf3D7_12_v3:1194287	ABC transporter, (CT family) (MRP2)	A1643V	**0.008064516**	**0.048387097**
Pf3D7_12_v3:1194623	ABC transporter, (CT family) (MRP2)	L1531P	0	0.032258065
Pf3D7_13_v3:171351	sodium/hydrogen exchanger, Na ,+H++antiporter (NHE)	I1480K	0	**0.046511628**
Pf3D7_13_v3:171587	sodium/hydrogen exchanger, Na ,+H++antiporter (NHE)	H1401Q	0.025974026	**0.046511628**
Pf3D7_13_v3:175170	sodium/hydrogen exchanger, Na ,+H++antiporter (NHE)	S207F	0	**0.097560976**
Pf3D7_13_v3:175627	sodium/hydrogen exchanger, Na ,+H++antiporter (NHE)	E55K	0.012820513	0.022727273
Pf3D7_13_v3:175648	sodium/hydrogen exchanger, Na ,+H++antiporter (NHE)	D48Y	0	0.022727273
Pf3D7_14_v3:1956918	multidrug resistance protein 2+(heavy metal transport family) (MDR2)	K253T	0	0.008064516
Pf3D7_14_v3:1956928	multidrug resistance protein 2+(heavy metal transport family) (MDR2)	V250I	0	0.032258065
Pf3D7_14_v3:297920	plasmepsin III,histo-aspartic protease (HAP)	K151N	0.008064516	0.024193548

Supplementary table 5: Primers and probes for Taqman allelic discrimination assays for K13 propeller domain nucleotide polymorphism that show strong association with the delayed clearance phenotype for artemisinin resistance.

Sequence name	5' Modification	Sequence	3' Modification
PFK13-SNP493Fwd		GCTGGCGTATGTGTACACCTATG	
PFK13-SNP493Rev		ACCTCAGTTTCAAATAAAGCCTTATAATC	
PFK13-SNP493Allele1	6-Fam	AATTTCTTA***C***ACGTTTTTGGTGG	BHQ-1
PFK13-SNP493Allele2	Hex	#ATTTCTTA***T***ACGTTTTTGGT	BHQ-1
PFK13-SNP539Fwd		TGAGGTGTATGATCGTTTAAGAGATGT	
PFK13-SNP539Rev		GCCATCATATCCCCCAATACAA	
PFK13-SNP539Allele1	6-Fam	TGTTACGTCAAATGGTA***C***AA	BHQ-1
PFK13-SNP539Allele2	Hex	TGTTACGTCAAATGGTA***G***AA	BHQ-1
PFK13-SNP543Fwd		GAAGAAATAATTGTGGTGTTACGTCAA	
PFK13-SNP543Rev		CATTCGGTATAATAGAAGAGCCATCA	
PFK13-SNP543Allele1	6-Fam	TGGTAGAATTTATTGTA***C***TGGG	BHQ-1
PFK13-SNP543Allele2	Hex	TGGTAGAATTTATTGTA***T***TGGG	BHQ-1
PFK13-SNP580Fwd		GCACCTTTGAATACCCCTAGATCA	
PFK13-SNP580Rev		ATCTCTCACCATTAGTTCCACCAAT	
PFK13-SNP580Allele1	6-Fam	CAGCTATGT***A***TGTTGC	BHQ-1
PFK13-SNP580Allele2	Hex	CAGCTATGT***G***TGTTGCTT	BHQ-1
PFK13-SNP543-Y-53FWd		CCACCTCTACCCATGCTTTCATAC	
PFK13-SNP543-Y-53Rev		GTGGTGTTACGTCAAATGGTAGAAT	
PFK13-SNP543-Y-53Allele1	6-Fam	TCATATCCCCCA***A***TACAATA	BHQ-1
PFK13-SNP543-Y-53Allele2	Hex	#CATATCCCCCA***G***TACAATA	BHQ-1
PFK13-SNP580-R-74Fwd		TGGCACCTTTGAATACCCCTA	
PFK13-SNP580-R-74Rev		AATCTCTCACCATTAGTTCCACCAA	
PFK13-SNP580-R-74Allele1	6-Fam	CATCAGCTATGT***A***TGTTGCT	BHQ-1
PFK13-SNP580-R-74Allele2	Hex	TCAGCTATGTGT***G***TTGCT	BHQ-1

Supplementary table 6: Fst and p-values (in parentheses) for all pairs of populations: Standard deviations of F-statistics were obtained through jack-knifing over loci. 95% confidence intervals of F-statistics were obtained through bootstrapping over loci.

Population	2013	2014	2015
**2013**	--		
**2014**	**0.007** *(0.099)*	--	
**2015**	**0.006** *(0.100)*	**0.006** *(0.159)*	--

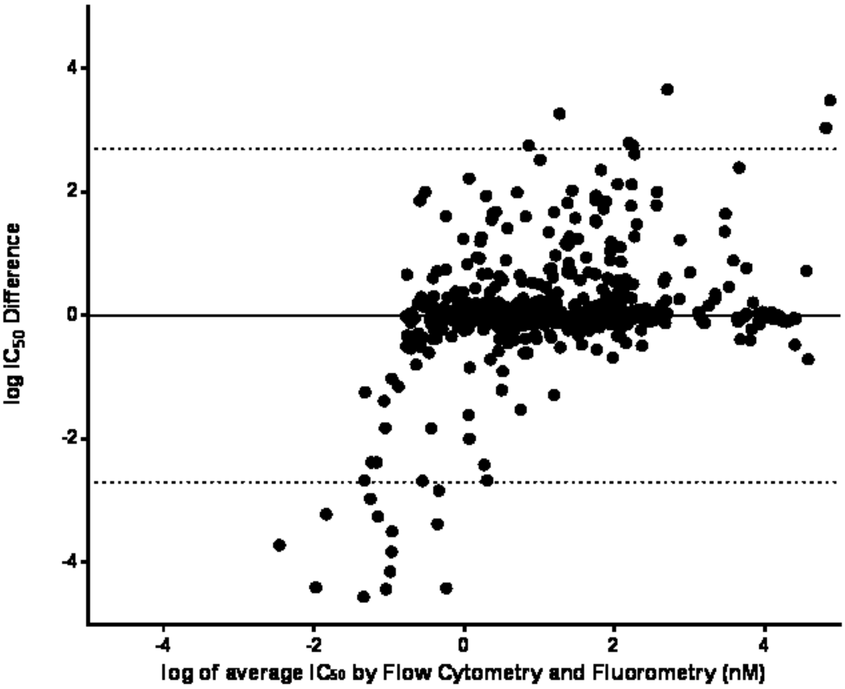

Supplementary figure 1; Bland-Altman plot of agreement between IC50s determined by curve fitting in Graphpad prism and IVART. Log transformed IC50 values were used to enable normality

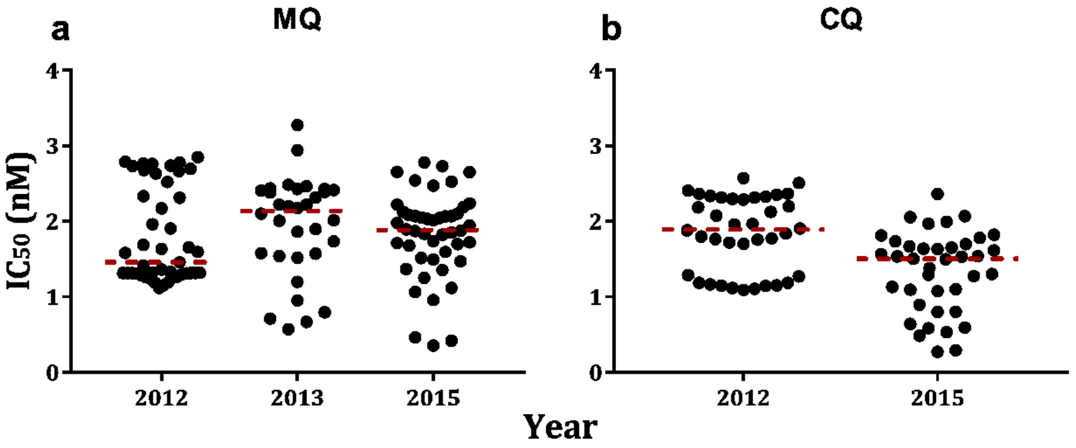

Supplementary figure 2: Temporal changes in mefloquine (MQ) and chloroquine (CQ) susceptibility (IC_{50}) of *P. falciparum* clinical isolates collected in 2012-2015 transmission season from Brikama (Western Gambia). Each plot shows the logIC_{50} per isolate for (a) MQ determined for 2012, 2013 and 2015 labelled on the x-axis and (b) CQ determined for 2012 and 2013. Broken red lines indicate the median for each yearly population.

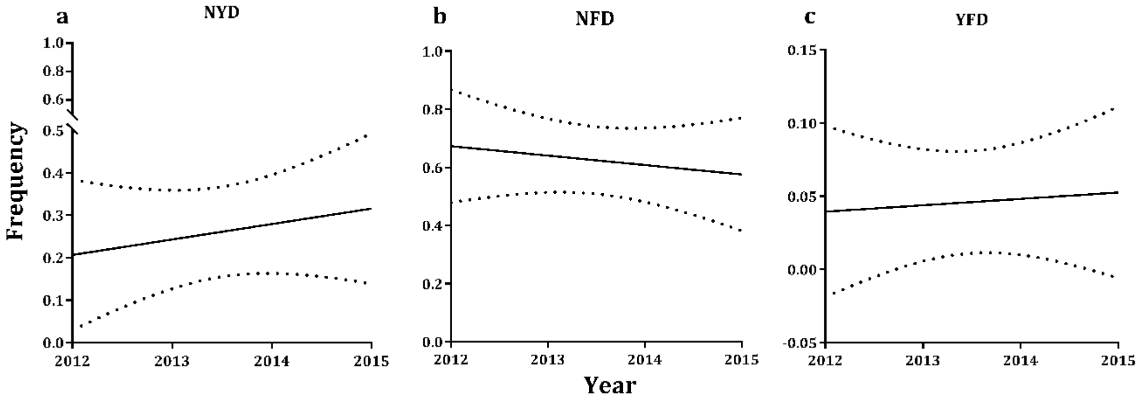

Supplementary figure 3: Linear regression fitting of proportion of haplotypes across transmission years. There was an increase in frequency of Pfmdr1 NYD (a) but decrease in frequency of NFD (b). (c) The YFD haplotype was at low frequency and was stable across the four transmission seasons.

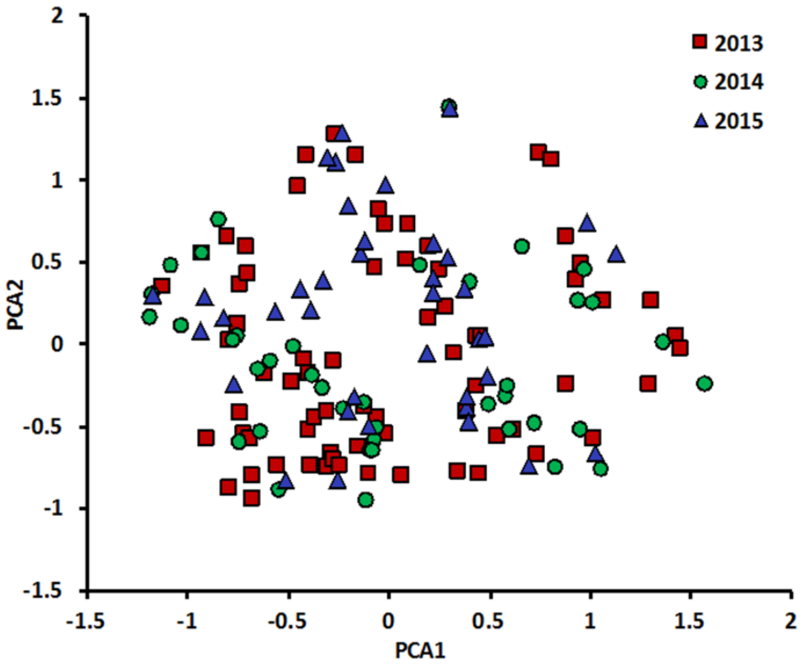

Supplementary figure 4: Scatter plot of principal components eigen values of axes 1 and 2 for all isolates analysed across the 3 transmission seasons (2013-2015). Marker types and colours are specific for each yearly population.
